# The severity of imiquimod-induced mouse skin inflammation is independent of endogenous IL-38 expression

**DOI:** 10.1371/journal.pone.0194667

**Published:** 2018-03-19

**Authors:** Jennifer Palomo, Sabina Troccaz, Dominique Talabot-Ayer, Emiliana Rodriguez, Gaby Palmer

**Affiliations:** 1 Division of Rheumatology, Department of Internal Medicine Specialties, University Hospitals of Geneva, Geneva, Switzerland; 2 Department of Pathology-Immunology, University of Geneva School of Medicine, Geneva, Switzerland; Northwestern University, UNITED STATES

## Abstract

The IL-1 cytokine family includes eleven members, among which Il-36α, β and γ, IL-36Ra and IL-38. The IL-36 cytokines are involved in the pathogenesis of psoriasis. IL-38 is also expressed in the skin and was previously proposed to act as an IL-36 antagonist. In this study, we thus examined expression and function of Il-38 in a mouse model of imiquimod (IMQ)-induced skin inflammation. *Il-38* mRNA was detected in the epidermis and in primary mouse keratinocytes, but not in dermal fibroblasts. At the peak of IMQ-induced inflammation, skin *Il-38* mRNA levels were reduced, whereas *Il-36ra* mRNA expression increased. The severity of IMQ-induced skin inflammation, as assessed by recording ear thickness and histological changes, was similar in Il-38 KO and WT littermate control mice, while, in contrast, Il-36ra-deficient mice displayed more severe skin pathology than their WT littermates. Il-38-deficiency had no impact on IMQ-induced expression of proinflammatory mediators in the skin *in vivo*, on the basal expression of various cytokines or chemokines by cultured primary keratinocytes and dermal fibroblasts *in vitro*, or on the response of these cells to Il-36β. Finally, after cessation of topical IMQ application, the resolution of skin inflammation was also not altered in Il-38 KO mice. In conclusion, Il-38-deficiency did not impact the development or resolution of IMQ-induced skin inflammation. Our observations further suggest that endogenous Il-38 does not exert Il-36 inhibitory activity in this model, or in cultured skin cells. A potential anti-inflammatory function of Il-38 in mouse skin thus still remains to be demonstrated.

## Introduction

Psoriasis is a chronic disease characterized by localized or generalized skin lesions including erythematous plaques and lamellar scales [1], [2]. In a significant proportion of patients, the skin disease is accompanied by arthritis [3]. To date, many aspects of psoriasis pathogenesis remain unclear, but a dysregulated crosstalk between immune and skin cells is believed to underlie epidermal hyper-proliferation and hyperplasia, leukocyte infiltration and vascular proliferation in the papillary dermis [4, 5]. Historically, studies first focused on immune cells, but recently, non-immune cells, in particular keratinocytes and fibroblasts, were shown to play important roles in the disease process [6]. Various environmental triggers can induce or exacerbate psoriasis in humans, among which imiquimod (IMQ), a Toll-Like Receptor (TLR)7 agonist that activates the innate immune response [7, 8]. Similarly, topical application of the IMQ-containing Aldara cream on mouse skin causes cutaneous inflammation with leukocyte influx and epidermal hyperplasia, resembling human psoriatic lesions [9–11].

The IL-1 family of cytokines includes seven agonists, IL-1α, IL-1β, IL-18, IL-33, IL-36α, IL-36β and IL-36γ, and four established or hypothetical antagonists, IL-1Ra, IL-36Ra, IL-37 and IL-38 [12, 13]. The three IL-36 agonists signal through the same receptor, composed of the specific alpha chain IL-36R (IL-1Rrp2) and the common beta chain IL-1 receptor accessory protein [14]. IL-36 cytokines and IL-36R are mainly expressed by keratinocytes, but also by dendritic cells and Th0 cells [15, 16]. IL-36 agonists are involved in the pathogenesis of skin inflammation both in mouse models and in human psoriasis [17]. In particular, in mice, IL-36 plays a crucial role in the development of IMQ-induced skin inflammation, which is exacerbated in absence of its antagonist IL-36Ra [15]. In humans, IL-36Ra deficiency results in a severe form of pustular psoriasis [18].

Conversely, little is known about IL-38, which was initially proposed to act as an antagonist based on its sequence homology with IL-1Ra and IL-36Ra [19]. Consistently, anti-inflammatory effects were subsequently reported for IL-38 in cultured cells [20–23] and in mice [23–26]. In humans, polymorphisms in the *IL1F10* locus are associated with rheumatic diseases [27–30] and IL-38 expression or serum levels have been recorded in some autoimmune pathologies [21, 24, 31–33], but overall few data are available concerning the role of IL-38 in inflammatory diseases. The identity of the IL-38 receptor(s) also remains elusive, although IL-1 receptor type I [19], IL-36R [20] and interleukin-1 receptor accessory protein-like 1 (IL1RAPL1, also named TIGIRR-2) [22] have successively been proposed as candidates. Recently, we observed reduced *IL-38* transcript levels in human psoriatic skin, whereas *IL-38* expression was increased in colonic biopsies of Crohn’s disease patients and in synovial tissues of patients suffering from rheumatoid arthritis [17].

Given the importance of the IL-36 system in the skin and since IL-38 was previously proposed to act as an IL-36R antagonist [20], in the present study we examined the expression and function of endogenous Il-38 in the context of IMQ-induced skin inflammation. We confirmed that *Il-38* was mainly expressed by keratinocytes in the mouse, as previously reported for human skin [19], but not by dermal fibroblasts. Furthermore, our data obtained using Il-38 KO mice indicate that, in contrast to Il-36ra-deficiency, lack of endogenous Il-38 does not impact the development or resolution of IMQ-induced skin inflammation.

## Materials and methods

### Mice

*Il-38* (*Il-1f10*)–deficient mice (*Il-38*^*-/-*^; Balb.129/Sv-Il1f10) and *Il-36ra* (*Il-1f5*)–deficient mice (*Il-36ra*^*-/-*^; Balb.B6.129S5/SvEv-Il36rn) [34] were created by Amgen Inc. (Seattle, WA, USA). *Il-38*^-/-^ mice were generated by targeting of the *Il1f10* gene in 129Sv ES GS1 cells, resulting in the deletion of all coding exons and leading to a complete loss of *Il-38* mRNA expression (S1 Fig). Genotyping of *Il-38*^-/-^ mice was performed using a 3-primer PCR combining a forward primer specific for the wild-type (WT) (5’-TGG CCC AGC TGA GCC CCA GCA GCC AGT-3’) or the KO (5’-CAG CTT CTG TTC CAC ATA CAC TTC-3’) allele with a common reverse primer (5’-TGC TGA GCA AGA AGA TCT CAG ACT-3’) (S1 Fig). Genotyping of *Il-36ra*^*-/-*^ mice was performed using a 3-primer PCR combining a forward primer specific for the WT (5’-GAA AAG AGA GAG TGA ATG GGA G-3’) or the KO (5’-GAT TGC ACG CAG GTT CTC-3’) allele with a common reverse primer (5’-GAG CTC CAT GAT GTT CAC TGG-3’). *Il-38* and *Il-36ra*-deficient mice were backcrossed onto the BALB/cJ background using a marker-assisted selection protocol (MASP). The purity of the BALB/cJ background, as assessed by genome-wide single nucleotide polymorphism (SNP) scanning using a 384 SNP panel with SNPs spread across the genome at 7 Mbp intervals (Charles River Laboratories, Wilmington, MA) was > 97% and > 99% for *Il-38*^*-/-*^ and *Il-36ra*^*-/-*^ mice respectively. For both mouse lines, heterozygous breedings were then set up to obtain *Il-38*^*-/-*^ or *Il-36ra*^*-/-*^ mice and their respective WT co-housed littermates for experiments. All mice were bred and maintained in the conventional area of the animal facility at the Geneva University School of Medicine and housed in open cages, enriched with Nordic aspen bedding (Tapvei, Harjumaa, Estonia), nestlets, and a mouse house, in groups of 2–6 individuals on a 12h light/dark cycle. The temperature in the room was maintained between 20–24° Celsius and hygrometry was 30–70%. Extruded food and tap water were provided *ad libitum*. Mice were monitored daily for signs of distress (signs of dehydration, unresponsiveness to extraneous stimuli, hunched posture, or labored breathing) and would have been euthanized should these signs have appeared. Animal studies were approved by the Animal Ethics Committee of the University of Geneva and the Geneva Veterinarian Office (authorizations GE-43-15 and GE-115-17) and complied with the requirements defined by the Swiss regulation (federal animal protection ordinances and law). Experiments were performed according to the appropriate codes of practice and all efforts were made to minimize suffering.

### Isolation of skin, epidermis and primary culture of keratinocytes and dermal fibroblasts

To harvest untreated skin from tails and ears for RNA extraction and for the isolation and culture of primary cells, naïve mice were euthanized by exposure to gradually increasing concentrations of carbon dioxide (CO_2_) in a dedicated euthanasia chamber. For the comparison of cytokine expression in naïve total skin and in the epidermis, a fragment of shaved abdominal skin was removed, rinsed in PBS / 100 U/ml penicillin / 100 μg/ml streptomycin and incubated in Keratinocyte-Serum Free Medium (K-SFM) (Life Technologies, Carlsbad, Ca, USA) / 10mg/ml Dispase II (Sigma-Aldrich, Saint-Louis, Mi, USA) overnight at 4°C. The piece of skin from each mouse was cut into halves. One part was immediately frozen in liquid N_2_, while, for the second part, the epidermis was detached from the dermis, collected and frozen. For keratinocyte culture, mouse tails were removed, rinsed in PBS / 100 U/ml penicillin / 100 μg/ml streptomycin and incubated in K-SFM / 10mg/ml Dispase II overnight at 4°C. Epidermis was then detached from the dermis and gently mixed 3 times for 1 minute with 0.05% Trypsin / 0.02% EDTA. Isolated cells were cultured in collagen type IV coated plates, in K-SFM complemented with 53.4 μg/ml Bovine Pituitary Extract (BPE) and 6.6 ng/ml human recombinant EGF (Life Technologies). The cells were used when they reached 80% confluence. For dermal fibroblast culture, ears were removed, minced and incubated for 2 h in HBSS / Ca^2+^ / Mg^2+^ / 2 mg/ml collagenase (Sigma-Aldrich) at 37°C. The tissue was then digested for 30 min in 0.05% Trypsin / 0.02% EDTA at 37°C and the cells and tissue pieces were cultured in Petri dishes in DMEM / 10% FBS / 1 x non-essential amino acids / 100 U/ml penicillin / 100 μg/ml streptomycin to recover fibroblasts, which were used after the third passage. Purity of the isolated epidermal fraction and of keratinocyte and fibroblast cultures was verified by analyzing mRNA expression of keratinocyte-specific *Keratin 14* and fibroblast-specific *Collagen 1a* and *Vimentin* markers (S2 Fig).

### IMQ-induced skin inflammation

Psoriasis-like skin inflammation was induced in adult, age-matched, 8 to 12-week-old female *Il-38*^*-/-*^ or *Il-36ra*^*-/-*^ mice and their respective WT littermates by daily application of a topical dose of 12.5mg of Aldara^™^ cream (Meda Pharma GmbH, Frankfurt, Germany), containing 5% (0.625mg) of imiquimod (IMQ), on one ear during 7–8 days. Body weight was recorded and ear thickness was measured daily using a pocket thickness gage (Mitutoyo Europe GmbH, Dusseldorf, Germany). At the end of the experiment, mice were euthanized under deep terminal anesthesia by exsanguination (cardiac puncture) followed by cervical dislocation. Ears were collected for histological analysis and RNA extraction.

### Histopathological evaluation and immunohistochemistry

Ears were fixed in 4% buffered formaldehyde and embedded in paraffin. Ear sections (4μm) were deparaffinized and stained with hematoxylin and eosin (HE; Diapath S.p.A., Milano, Italy). Ly6G, CD3 and B220 expression was examined by immunohistochemistry on paraffin sections using the following antibodies: rat anti-mouse Ly6G (clone 1A8, BD Bioscience, 1/1000), rat anti-human CD3 (clone CD3-12, AbD Serotec, Kidlington, UK, 1/200), or rat anti-mouse B220 (clone RA3-6B2, BD Bioscience, 1/200). Tissue sections were deparaffinized and antigens retrieved by pressure-cooking in 10 mM citrate buffer, pH 6 (anti-Ly6G or anti-B220 staining) or in 10 mM Tris, 1 mM EDTA buffer, pH 9 (anti-CD3 staining). Slides were blocked for endogenous peroxidase activity and incubated with anti-Ly6G, CD3 or B220 antibodies in antibody diluent (S2022, Dako AG, Baar, Switzerland) overnight at 4°C. Subsequently, slides were incubated with appropriate HRP-conjugated secondary antibodies in antibody diluent and developed with diaminobenzidine (Dako). Slides were scanned on a Mirax Midi slide scanner (Carl Zeiss Microscopy, Feldbach, Switzerland). The ZEN blue software (Carl Zeiss Microscopy) was used for image acquisition and measurements. Total ear area was determined on HE-stained sections using the Definiens Developer XD2 software (Definiens, Munich, Germany) and different histopathological parameters were determined in a blinded manner. The average epidermal thickness was estimated by taking 20 measures along the ear. Infiltration of inflammatory cells was evaluated using a modification of the semi-quantitative analysis described previously [9], in which we evaluated the proportion of ear tissue containing infiltrated neutrophils, instead of using scores to reflect differential cell counts. Neutrophils infiltrating the dermis were identified morphologically on HE-stained sections. Areas containing infiltrated neutrophils were then delineated manually and the sum of all neutrophil-containing areas was normalized to the total ear surface. Consistent with previous reports [9, 35], we also observed neutrophil-filled abscess-like structures beneath the stratum corneum in IMQ-treated mice, which were identified morphologically on HE-stained sections and counted manually along the whole ear.

### RNA extraction and RT qPCR

Total RNA was extracted using TRIzol^®^ reagent (Life Technologies) and treated with RNAse free DNAse set (Qiagen, Hilden, Germany) according to the manufacturer's instructions. Total RNA (100-500ng) was then reverse transcribed using SuperScript II Reverse transcriptase (Invitrogen, Waltham, USA). The mRNA expression levels were determined by quantitative PCR using the SYBR^®^ Green PCR Master Mix (Applied Biosystem, Waltham, USA) according to the manufacturer’s protocol. The primer sequences (Eurofins, Ebersberg, Germany) are detailed in Table 1. Relative levels of mRNA expression were normalized to ribosomal protein L32 (*Rpl32*) mRNA levels using a comparative method (2^-ΔCt^). Non-reverse-transcribed RNA samples and Buffer were included as negative controls.

**Table 1:** Primers used for qPCR.

Gene		Accession number	Primer sequence	Amplicon (pb)
*Col1a1*		NM_007742.4	Fwd 5’-GGCTCCTGCTCCTCTTAG-3’ Rev 5’-ACAGTCCAGTTCTTCATTGC-3’	194
*Cxcl1*		NM_008176.3	Fwd 5'-ACTCAAGAATGGTCGCGAGG-3' Rev 5'-GTGCCATCAGAGCAGTCTGT-3’	123
*K14*	Var1	NM_016958.2	Fwd 5’-ATCGAGGACCTGAAGAGCAA-3’ Rev 5’-GGCTCTCAATCTGCATCTCC-3’	220
	Var2	NM_001313956.1		
*Il-1a*		NM_010554.4	Fwd 5'-GGGAAGATTCTGAAGAAGAG-3' Rev 5'-GAGTAACAGGATATTTAGAGTCG-3’	319
*Il-1b*		NM_008361.4	Fwd 5'-TGTGAAATGCCACCTTTTGA-3' Rev 5'-GTGCTCATGTCCTCATCCTG-3’	248
*Il-6*		NM_031168.2	Fwd 5'-TGAACAACGATGATGCACTTGCAGA-3' Rev 5'-TCTGTATCTCTCTGAAGGACTCTGGCT-3’	211
*Il-18*		NM_008360.1	Fwd 5'-CAGGCCTGACATCTTCTG-3' Rev 5'-CTGACATGGCAGCCATT-3’	104
*Il-36a*		NM_019450.3	Fwd 5'-TAGTGGGTGTAGTTCTGTAGTGTGC-3' Rev 5'-GTTCGTTCAAGAGTGTCCAGATAT-3’	268
*Il-36b*		NM_027163.4	Fwd 5'-ACAAAAAGCCTTTCTGTTCTATCAT-3' Rev 5'-CCATGTTGGATTTACTTCTCAGACT-3’	186
*Il-36g*		NM_153511.3	Fwd 5'-AGAGTAACCCCAGTCAGCGTG-3' Rev 5'-AGGGTGGTGGTACAAATCCAA-3’	186
*Il-36r*		NM_133193.3	Fwd 5'-AAACACCTAGCAAAAGCCCAG-3' Rev 5'-AGACTGCCCGATTTTCCTATG-3’	262
*Il-36ra*		NM_019451.2	Fwd 5'-TGGAGCTCATGATGGTTCTG-3' Rev 5'-TAATGACCTTCTCTGCGTGC-3’	123
*Il-38*		NM_153077.2	Fwd 5'-CCTGGCGTGTGTAAAGACAA-3' Rev 5'-CAGATCCCAAGCTTCTCTGG-3’	125
*Rpl32*		NM_172086.2	Fwd 5'-CACCAGTCAGACCGATATGTGAAAA-3' Rev 5'-TGTTGTCAATGCCTCTGGGTTT-3'	64
*S100a9*	Var1	NM_001281852.1	Fwd 5'-CACCCTGAGCAAGAAGGAAT-3' Rev 5'-TGTCATTTATGAGGGCTTCATTT-3'	95
	Var2	NM_009114.3		
*Tnfa*		NM_013693.3	Fwd 5'-AGTTCTATGGCCCAGACCCT-3' Rev 5'-GTCTTTGAGATCCATGCCGT-3’	159
*Vim*		NM_011701.4	Fwd 5’-CGGCTGCGAGAGAAATTGC -3’ Rev 5’-CCACTTTCCGTTCAAGGTCAAG-3’	124

### Statistical analysis

Data were analyzed using Prism version 6 (Graphpad Software, La Jolla, USA). Unpaired Mann-Whitney comparison tests, two-way ANOVA followed by a Holm–Sidak’s comparison test, or paired two-way ANOVA followed by a Sidak post-test were used, as indicated. Values are expressed as mean ± SEM. Statistical significance was defined at a p-value < 0.05.

## Results

### Expression of Il-38 and of Il-36 family cytokine mRNA in naïve mouse skin and in primary mouse skin cells

**Fig 1:**
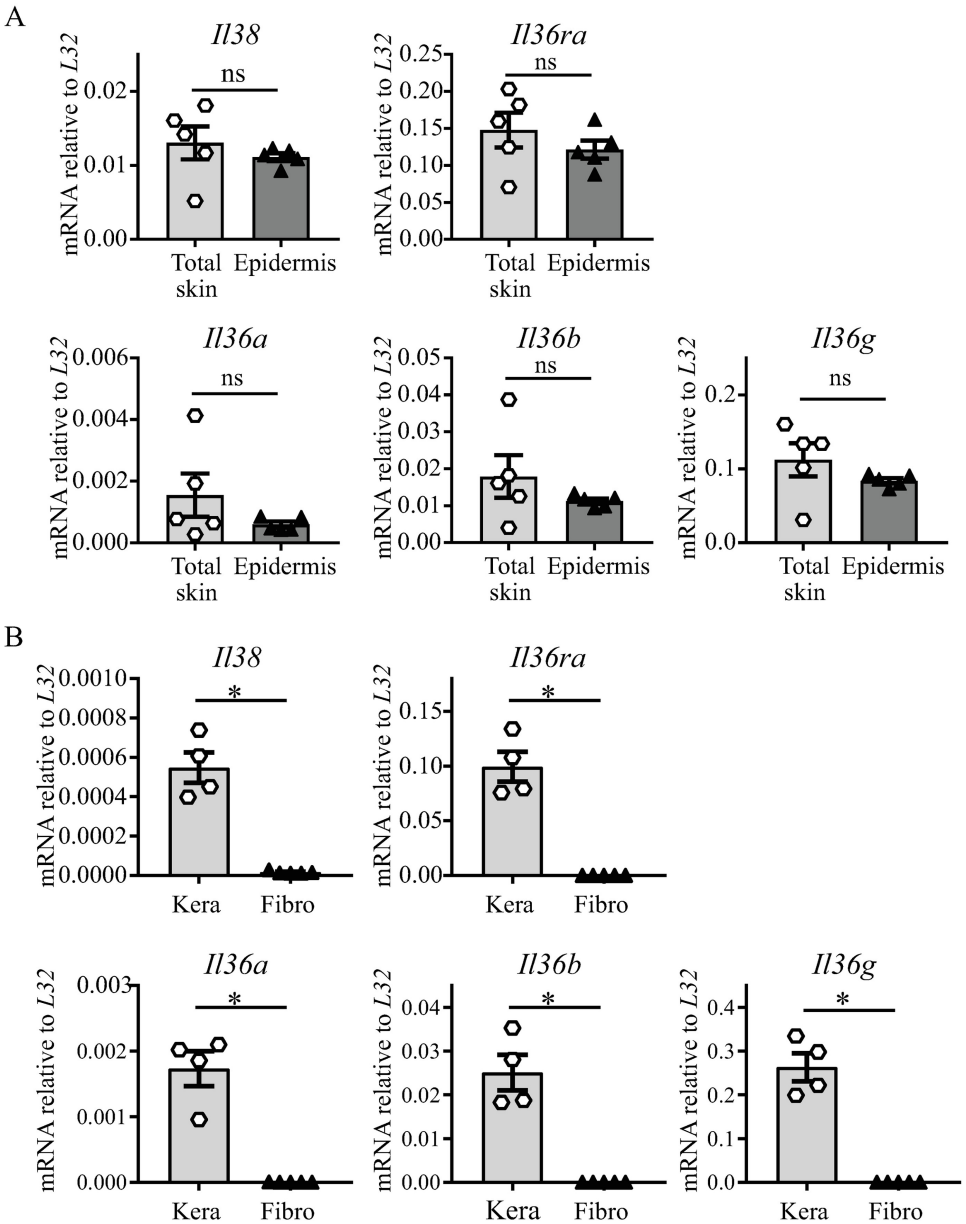
Expression of *Il-38* and *Il-36* family cytokines in total skin, epidermis, cultured primary keratinocytes and dermal fibroblasts. Basal *Il-38*, *Il-36ra*, *Il-36a*, *Il-36b* and *Il-36g* mRNA expression was quantified by real-time RT-qPCR in total skin (n = 5) and epidermis (n = 5) of naïve BALB/c WT mice (A); and in cultured primary keratinocytes (Kera, n = 4 independent cultures) and dermal fibroblasts (Fibro, n = 5 independent cultures) isolated from naïve BALB/c WT mice (B). Data were expressed relative to *L32* levels. Results are shown as individual values and mean ± SEM. Statistical analysis was performed using an unpaired Mann-Whitney comparison test. A *p*-value < 0.05 was considered significant. *** p<0.001, ** p<0.01, * p<0.05.

Keratinocytes express various pattern recognition receptors and act as early detectors of microbial or endogenous danger signals. After activation, they secrete chemokines, cytokines and anti-microbial peptides. In human skin, keratinocytes were suggested to be the main source of IL-38 [19]. In mouse skin, we previously observed *Il38* mRNA expression [17], but its cellular source had not been described. We first examined the mRNA expression of *Il-38* and of the different *Il-36* agonists and antagonist in total skin and isolated epidermis of naïve BALB/c mice. We detected similar levels of *Il-38*, *Il-36ra*, *Il-36a*, *Il-36b* and *Il-36g* mRNA in total skin and in epidermis (Fig 1A). We further investigated the expression of *Il-38* and of the *Il-36* agonists and antagonist in cultured primary keratinocytes and dermal fibroblasts isolated from naïve BALB/c mouse skin. We observed *Il-38* mRNA expression in keratinocytes, but not in dermal fibroblasts. Similarly, transcripts for *Il-36* agonists and *Il-36ra* were detected in keratinocytes only (Fig 1B). In contrast, Il-36 receptor (*Il-36r*) mRNA expression was observed in both skin cell types (Part B in S2 Fig).

### Il-38 deficiency has no impact on the development of IMQ-induced psoriasis

Consistent with our previous observations during the development of IMQ-induced skin inflammation in C57BL/6 mice [17], we confirmed decreased *Il-38* mRNA expression at the peak of IMQ-induced inflammation in the skin of BALB/c mice, whereas the mRNA levels of *Il-36ra* and of the *Il-36* agonists were increased after IMQ treatment (S3 Fig).

**Fig 2:**
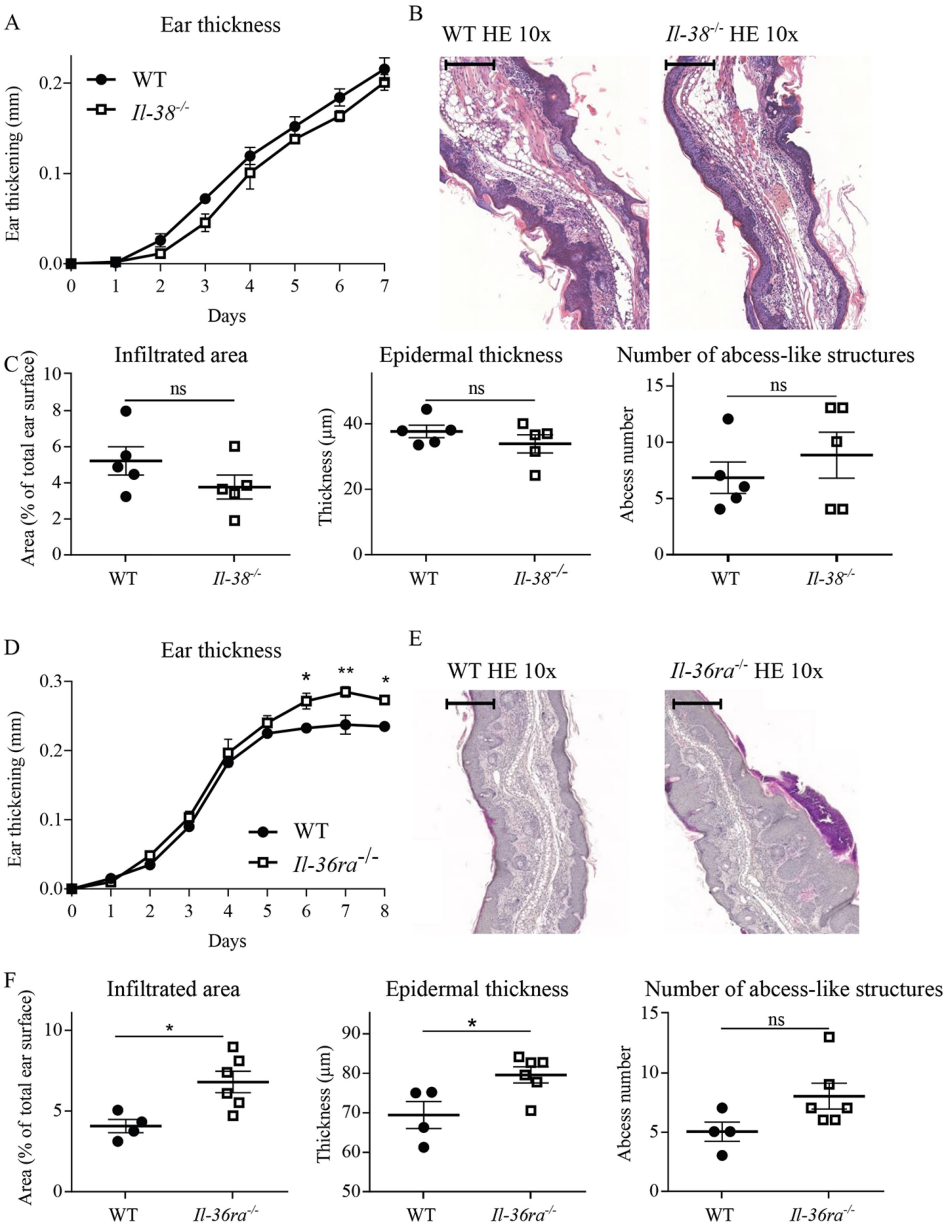
Il-38-deficiency does not influence the severity of IMQ-induced skin inflammation. *Il-38*^*-/-*^ mice (n = 5) and WT littermates (n = 5) were treated daily with a topical dose of 12.5mg of Aldara^™^ cream (0.625mg IMQ), for 7 days. Ear thickness was followed daily (A) and expressed as ear thickness variation vs. day 0. Microscopic histopathology was studied on HE-stained slides of IMQ-treated ears on day 7. Scale bar = 100μm. (B); neutrophil-infiltrated areas, epidermal thickness and the number of neutrophil-filled abscess-like structures were evaluated (C). Results are from one experiment representative of two and are expressed as mean ± SEM of individual mice (n = 5 mice per group). *Il-36ra*^*-/-*^ mice (n = 6) and WT littermates (n = 4) were treated daily with a topical dose of 12.5mg of Aldara^™^ cream (0.625mg IMQ), for 8 days. Ear thickness was followed daily (D) and expressed as ear thickness variation vs. day 0. Microscopic histopathology was studied on HE-stained slides of IMQ-treated ears on day 8. Scale bar = 100μm. (E); neutrophil-infiltrated areas, epidermal thickness and the number of neutrophil-filled abscess-like structures were evaluated (F). Results are expressed as mean ± SEM of individual mice (n = 4–6 mice per group). Statistical analysis was performed using a paired two-way ANOVA followed by a Sidak post-test for A and and D, and an unpaired Mann-Whitney comparison test in C and F. A *p*-value < 0.05 was considered significant. ** p<0.01, * p<0.05.

We then went on to investigate the involvement of endogenous Il-38 in the pathogenesis of IMQ-induced skin inflammation, using Il-38-deficient mice (S1 Fig). Homozygous *Il-38*^*-/-*^ mice are healthy, fertile, and show weight gain similar to that of their WT littermates from birth to adult age. They do not display any spontaneous phenotype in our conventional animal facility. *Il-38*-deficiency had no effect on the severity of IMQ-induced skin inflammation (Fig 2). Ear thickness increased similarly after IMQ application in *Il-38*^*-/-*^ mice and in their WT littermates (Fig 2A), and both groups of mice displayed comparable histopathological alterations on day 7 of IMQ-treatment (Fig 2B). Immunohistochemical analyses confirmed infiltration of IMQ-treated ears by inflammatory cells, in particular neutrophils, as reported previously [9, 35]. Abundant infiltration of Ly6G^+^ cells was observed predominantly in the dermis (Part A in S4 Fig). In addition, characteristic neutrophil-filled abscess-like structures were found just beneath the stratum corneum [35] (Part B in S4 Fig). CD3^+^ T cells were detected both in the dermis and the epidermis, while some infrequent B220^+^ B lymphocytes were detected in the dermis exclusively (Part A in S4 Fig). We did not observe any qualitative differences in infiltrate composition between WT and Il-38 KO mice, as illustrated by anti-Ly6G and anti-CD3 staining of ear sections after 7 days of IMQ-treatment (Part C in S4 Fig). Furthermore, histopathological scoring indicated that the extent of neutrophil infiltration, the epidermal thickness and the numbers of neutrophil-filled abscess-like structures were similar in *Il-38*^*-/-*^ mice and in their WT littermates (Fig 2C).

We compared these observations with the response of mice deficient in Il-36Ra, a well-known inhibitor of IL-36-dependent IMQ-induced skin inflammation [15]. We confirmed that *Il-36ra*-deficiency resulted in an aggravation of skin pathology. Indeed, *Il-36ra*^*-/-*^ mice developed a more severe disease, as shown by an increased ear thickening, as compared to their WT littermates (Fig 2D). This was associated with more severe histopathological changes (Fig 2E and 2F).

### IL-38 deficiency has no impact on IMQ-induced expression of proinflammatory mediators *in situ* or in cultured skin cells

**Fig 3:**
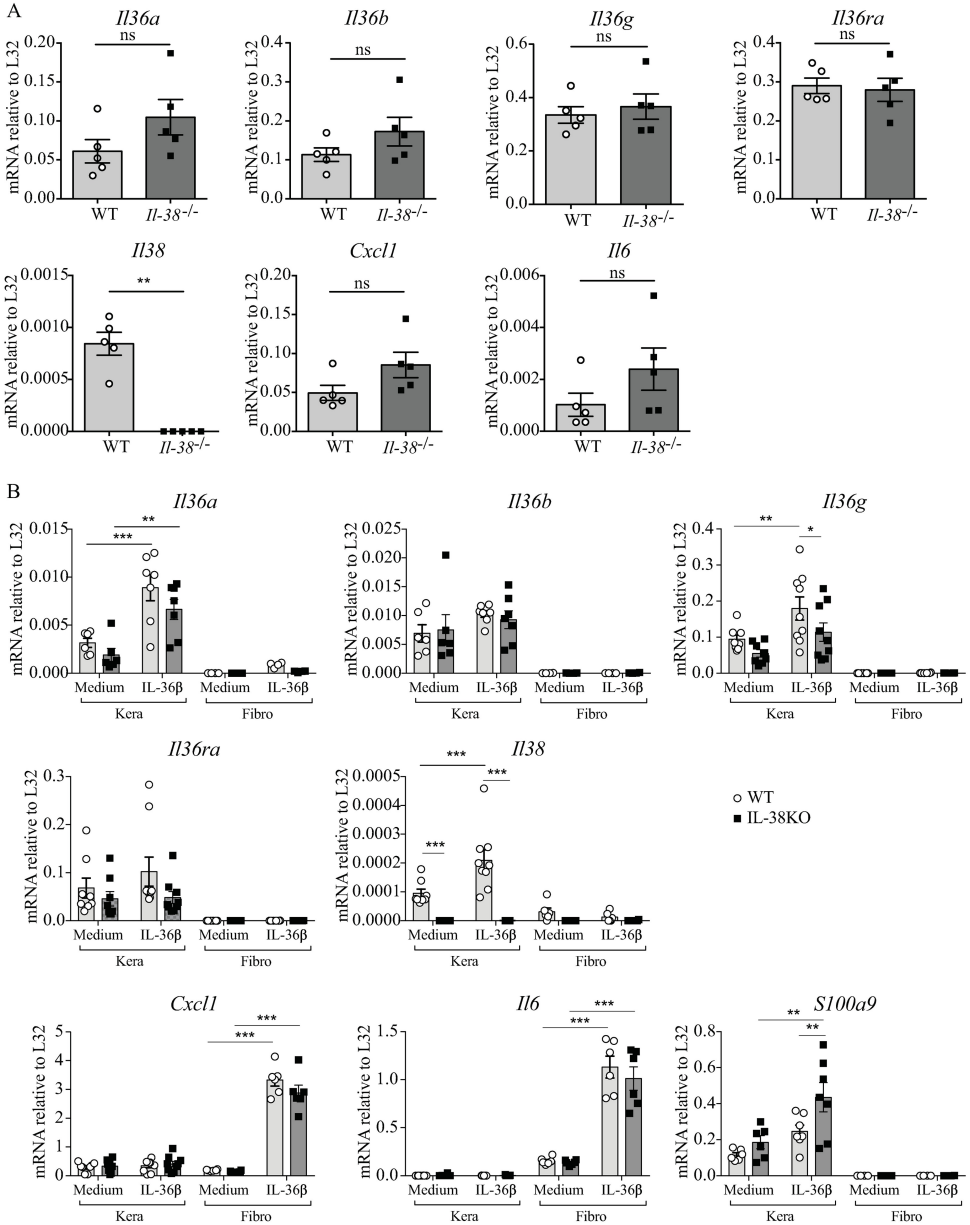
Expression of proinflammatory mediators in IMQ-treated skin and in cultured skin cells of Il-38 deficient mice. *Il-38*^*-/-*^ mice and WT littermates were treated daily with a topical dose of 12.5mg of Aldara^™^ cream (0.625mg IMQ), for 7 days. Skin mRNA levels for *Il-36α*, *Il-36β*, *Il-36γ*, *Il-36ra*, *Il-38*, *Cxcl-1 and Il-6* were quantified by real-time RT-qPCR on day 7 in the IMQ-treated ears (A). Data were expressed relative to *L32* levels. Results represent individual values and mean ± SEM of n = 5 mice per group. Statistical analysis was performed by unpaired Mann-Whitney comparison test. A *p*-value < 0.05 was considered significant. ** p<0.01. Cultured primary keratinocytes (Kera) and dermal fibroblasts (Fibro) isolated from naïve *Il-38*^*-/-*^ (dark symbols) or WT mice (white symbols), were stimulated with rec. mouse Il-36β at 100ng/ml for 6 h, or left unstimulated (Med). *Il-36α*, *Il-36β*, *Il-36γ*, *Il-36ra*, *Il-38*, *Cxcl-1*, *Il-6* and *S100a9* mRNA levels were quantified by real-time RT-qPCR (B). Data are expressed relative to *L32* levels. Results represent individual values and mean ± SEM of n = 6–9 biological replicates per group. Statistical analysis was performed by two-way ANOVA followed by a Holm–Sidak’s comparison test. A *p*-value < 0.05 was considered significant. *** p<0.001, ** p<0.01, * p<0.05.

We examined whether the lack of endogenous *Il-38* could nevertheless influence the local expression of proinflammatory mediators in the skin. Thus, we analyzed mRNA expression of various cytokines and chemokines in the ear after 7 days of IMQ application. We did not find any significant differences in *Il-36α*, *Il-36β*, *Il-36γ*, *Il-36ra*, *Cxc-l1*, *Il-6*, *Il-1α*, *IL-1β*, *Il-18* or *Tnfα* mRNA expression between *Il-38*^*-/-*^ and WT mice, while *Il-38* was obviously not expressed in *Il-38*^*-/-*^ mice (Fig 3A and S5 Fig**)**.

We further investigated the expression of proinflammatory mediators by cultured primary keratinocytes and dermal fibroblasts isolated from *Il-38*-deficient and WT mice, at baseline and upon stimulation with rec. mouse Il-36β. Although both cell types express *Il36r* (Part B in S2 Fig), keratinocytes and fibroblasts displayed differential responses to Il-36β. Indeed, in keratinocytes, Il-36β enhanced mRNA expression of *Il-36α*, *Il-36γ* and *Il-38*, and of the anti-microbial peptide *S100a9* (Fig 3B), while stimulation of dermal fibroblast with Il-36β strongly induced expression of *Il-6* and *Cxcl-1*. Basal expression levels of the various transcripts examined did not differ significantly in cells isolated from *Il-38*^*-/-*^ or from WT mice, except for the expression of *Il-38* itself. Il-38 deficiency also lacked any major impact on the response of cultured keratinocytes or fibroblasts to Il-36β (Fig 3B).

### IL-38 deficiency does not alter IMQ-induced psoriasis resolution

**Fig 4:**
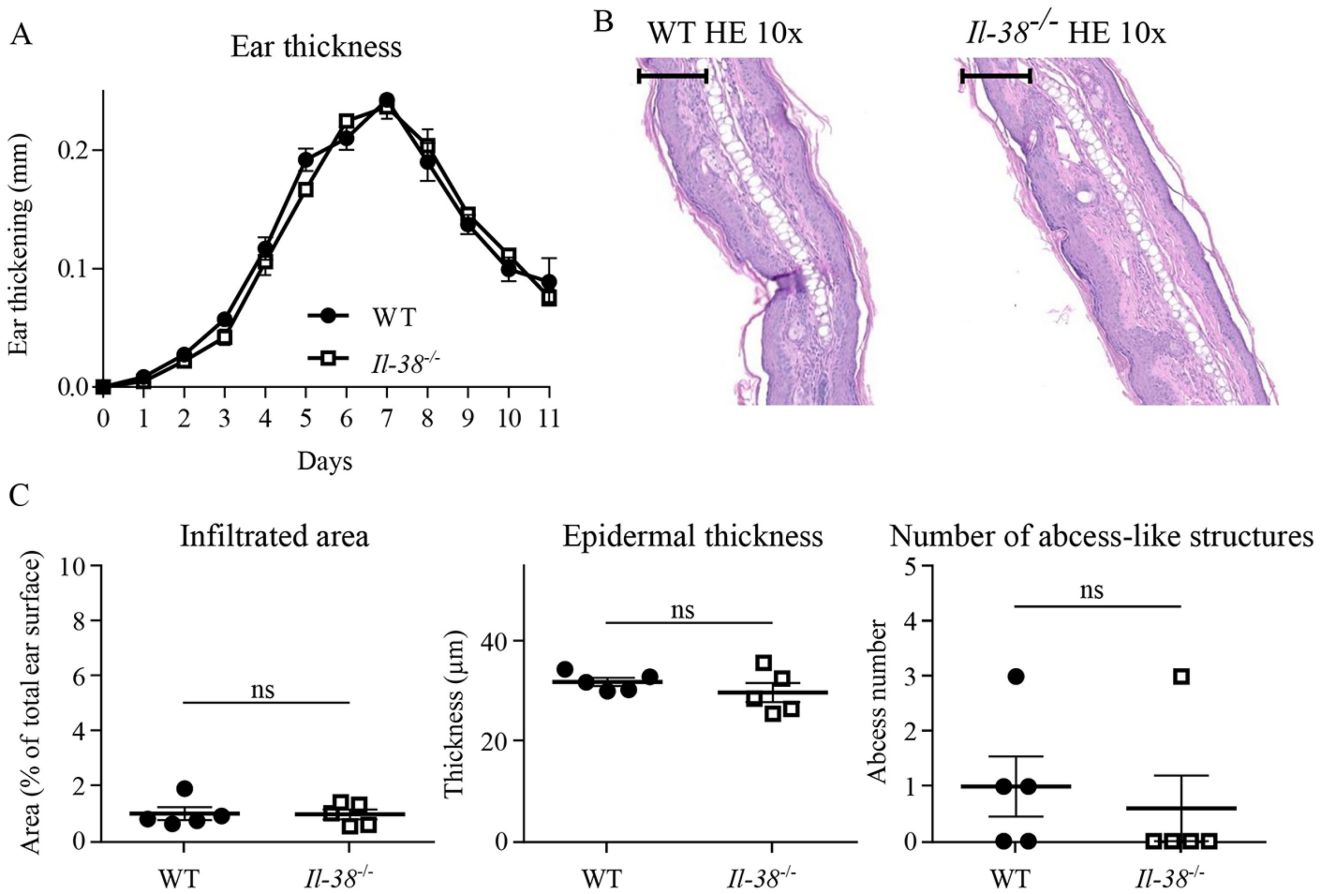
Il-38-deficiency does not affect resolution of IMQ-induced inflammation. *Il-38*^*-/-*^ mice (n = 5) and WT littermates (n = 5) were treated daily with a topical dose of 12.5mg of Aldara^™^ cream (0.625mg IMQ), for 7 days, then left untreated until day 11. Ear thickness was followed daily (A) and expressed as ear thickness variation vs. day 0. Microscopic histopathology was studied on HE-stained slides of IMQ-treated ears at day 11. Scale bar = 100 μm. (B); neutrophil-infiltrated areas, epidermal thickness and the number of neutrophil-filled abscess-like structures were evaluated (C). Results are representative of 2 independent experiments and expressed as mean ± SEM of individual mice (n = 5 mice per group). Statistical analysis was performed using a paired two-way ANOVA followed by a Sidak’s post-test for A and an unpaired Mann-Whitney comparison test in C. A *p*-value < 0.05 was considered significant.

Although Il-38 was not required for the development of IMQ-induced skin inflammation, we wondered whether it might still be involved in the resolution of the pathology. To answer this question, after 7 days of IMQ topical application, *Il-38*^*-/-*^ mice and their WT littermates were kept untreated for 5 days. As in Fig 2, the severity of peak skin inflammation was again similar in *Il-38*^*-/-*^ and WT mice. Afterwards, the gradual decrease of ear thickness was also similar in *Il-38*^*-/-*^ mice and in their WT littermates (Fig 4A). Those results were further confirmed by histological analysis. Indeed, neutrophil infiltration, epidermal thickness, and the number of neutrophil-filled abscess-like structures were comparable on day 11 in the presence or in the absence of Il-38 (Fig 4B and 4C).

## Discussion

IL-36 cytokines are involved in the pathogenesis of psoriasis [36], as well as in the development of IMQ-induced skin inflammation in the mouse [15, 34]. Since IL-38 is also expressed in the skin [17] and was previously proposed to act as an IL-36 antagonist [20], we studied the expression and function of Il-38 in the IMQ model. We detected *Il-38* mRNA expression in mouse epidermis and in primary mouse keratinocytes, consistent with expression of *IL-38* in keratinocytes in human skin [17, 19], but not in dermal fibroblasts. In agreement with our recent observations in psoriatic human skin [17], *Il-38* mRNA levels were decreased in mouse during skin inflammation *in vivo*. However, lack of endogenous *Il-38* did not impact the development or resolution of IMQ-induced psoriasis. In our hands, Il-38-deficiency did also not change expression of proinflammatory mediators in inflamed skin *in situ* or in cultured skin cells, nor modify the response of primary keratinocytes and dermal fibroblasts to IL-36 stimulation.

Our *in vivo* data indicate that, in contrast to Il-36ra-deficiency, the absence of Il-38 does not impact the course of IMQ-induced skin inflammation, implying that endogenous Il-38 does not act as an Il-36 antagonist in this context. As it has been suggested that the anti-inflammatory properties of IL-38 are inferior as compared to IL-36Ra [20], it is conceivable that Il-38 deficiency is counterbalanced by the presence of Il-36Ra, whose role was confirmed in this study. Alternatively, the biological function of IL-38 might be unrelated to IL-36 inhibition. Several recent studies indeed demonstrated broader anti-inflammatory properties of IL-38 and/or suggested different mechanisms of action [20–26]. In contrast to several of these studies based on overexpression or injection of recombinant exogenous Il-38 [21, 23–26], we were not able to detect any anti-inflammatory, or other, activity of the endogenous protein in our model.

We further showed that, similarly to what was observed in human keratinocytes [17], murine keratinocytes, but not dermal fibroblasts, express IL-36 agonists, as well as IL-36Ra and IL-38. However, both keratinocytes and dermal fibroblasts expressed the Il36r, although, interestingly, the two cell types responded in a different way to IL-36. Indeed, keratinocytes rather amplified the IL-36 signaling by upregulating Il-36α expression, while fibroblasts produced pro-inflammatory mediators, such as Il-6 and the neutrophil-attracting chemokine Cxcl1. This is consistent with the role of keratinocytes as skin sentinels, which can detect early skin damage and release danger signals and pro-inflammatory mediators. This primary response can then be strongly amplified by dermal fibroblasts, which produce signals to recruit and activate immune cells. Since IL-38 was previously described to antagonize the effects of IL-36 stimulation [20], we also investigated the effects of Il-36 stimulation on primary skin cells isolated from WT and *Il-38*^-/-^ mice. However, Il-38-deficiency did not influence the response of primary keratinocytes or dermal fibroblasts to Il-36. Consistent with our *in vivo* observations, these *in vitro* results thus again failed to provide any evidence for an Il-36 inhibitory function of endogenous Il-38.

In conclusion, while this study does not exclude an inhibitory role of IL-38 in other contexts, our results indicate that Il-38-deficiency does not impact the development or resolution of IMQ-induced skin inflammation. Our observations further suggest that endogenous Il-38 does not exert Il-36 inhibitory activity in this model, or in cultured skin cells. An anti-inflammatory function, or any other role, of Il-38 in mouse skin thus still remain to be demonstrated.

## Supporting information

S1 Fig Generation of mice deficient for IL-38. Schematic representation of *Il1f10* gene invalidation: in the targeted allele, a neomycin selection cassette was inserted to replace all coding exons of the *Il1f10* gene (A). Mouse genotyping was performed on total DNA extracted from ear biopsies. PCR products for the WT (150 bp) and KO (250 bp) alleles are shown in *Il-38*^-/-^, *Il-38*^+/-^ and *Il-38*^+/+^ (WT) DNA samples (B). *Il-38* mRNA levels were quantified by real-time RT-qPCR on skin samples from naïve *Il-38*^-/-^ and WT mice. Data are expressed relative to *L32* levels. Results represent individual values and mean ± SEM of n = 3 per group (C).
(PDF)

S2 Fig Expression of keratinocyte and fibroblast specific markers in total skin, epidermis and primary skin cells. Basal mRNA expression of keratinocyte-specific *Keratin 14*, as well as of fibroblast-specific *Collagen 1a* and *Vimentin* was quantified by real-time RT-qPCR in total skin (n = 5) and epidermis (n = 5) of naïve BALB/c WT mice (A). *Keratin 14*, *Collagen 1a*, *Vimentin*, and *Il-36r* mRNA levels were quantified by real-time RT-qPCR in cultured primary skin keratinocytes (Kera, n = 4 independent cultures) and dermal fibroblasts (Fibro, n = 5 independent cultures) isolated from the skin of naïve WT BALB/c mice (B). Data are expressed relative to *L32* levels. Results represent individual values and mean ± SEM. Statistical analysis was performed using an unpaired Mann-Whitney comparison test. A *p*-value < 0.05 was considered significant. *** p<0.001, ** p<0.01, * p<0.05.
(PDF)

S3 Fig Expression of IL-38 and IL-36 family members in IMQ-treated skin of WT BALB/c mice. WT BALB/c mice were treated daily with a topical dose of 12.5mg of Aldara^™^ cream (0.625mg IMQ) for 8 days (n = 7). Skin mRNA levels for *Il-38*, *Il-36ra*, *Il-36α*, *Il-36β* and *Il-36γ* were quantified by real-time RT-qPCR in the non-treated ear (Ctr) and in the IMQ-treated ear on day 8. Data were expressed relative to *L32* levels. Results represent individual values and mean ± SEM. Statistical analysis was performed by unpaired Mann-Whitney comparison test. A *p*-value < 0.05 was considered significant. ** p<0.01, *** p<0.001.
(PDF)

S4 Fig Characterization of the inflammatory infiltrate in IMQ-treated ears. Representative HE (upper left panel), anti-Ly6G (brown staining, upper right panel), anti-CD3 (brown staining, lower left panel), and anti-B220 (brown staining, arrows, lower right panel) stained sections are shown for IMQ-treated WT ears at the peak of inflammation on day 7 (A). Representative anti-Ly6G (brown staining, left panel) and HE (right panel) stained sections including neutrophil-filled abscess-like structures located just beneath the stratum corneum (arrows) are shown for IMQ-treated WT ears at the peak of inflammation on day 7 (B). Representative anti-Ly6G (brown staining, left panels) and anti-CD3 (brown staining, right panels) stained sections are shown for IMQ-treated ears of WT (upper panels) or *Il-38*^*-/-*^ (lower panels) littermate mice at the peak of inflammation on day 7 (C). Scale bar = 100 μM.
(PDF)

S5 Fig IMQ-induced expression of proinflammatory mediators in the skin of *Il-38* deficient mice. *Il-38*^*-/-*^ mice and WT littermates were treated daily with a topical dose of 12.5mg of Aldara^™^ cream (0.625mg IMQ), for 7 days (n = 5). Skin mRNA levels for *Il-1α*, *Il-1β*, *Il-18* and *Tnfa* were quantified by real-time RT-qPCR on day 7. Data were expressed relative to *L32* levels. Results represent individual values and mean ± SEM. Statistical analysis was performed by unpaired Mann-Whitney comparison test. No significant differences were observed between the groups.
(PDF)

## Abbreviations

IMQ: imiquimod
