# Supplementary figures and images for: The severity of imiquimod-induced mouse skin inflammation is independent of endogenous IL-38 expression
Source: PLoS One. 2018 Mar 19;13(3):e0194667. doi: 10.1371/journal.pone.0194667 (PMC5858842; doi:10.1371/journal.pone.0194667)

Figure S1

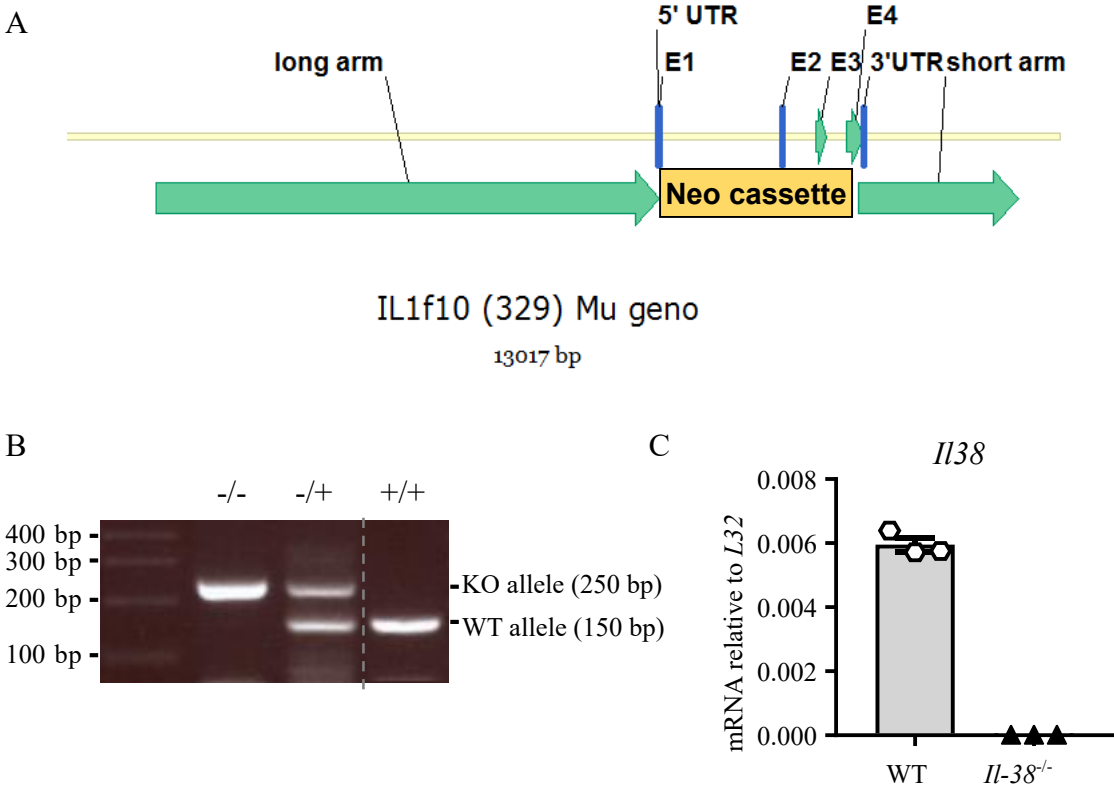

Supplement: S1 Fig — Schematic representation of Il1f10 gene invalidation: in the targeted allele, a neomycin selection cassette was inserted to replace all coding exons of the Il1f10 gene (A). Mouse genotyping was performed on total DNA extracted from ear biopsies. PCR products for the WT (150 bp) and KO (250 bp) alleles are shown in Il-38-/-, Il-38+/- and Il-38+/+ (WT) DNA samples (B). Il-38 mRNA levels were quantified by real-time RT-qPCR on skin samples from naïve Il-38-/- and WT mice. Data are expressed relative to L32 levels. Results represent individual values and mean ± SEM of n = 3 per group (C). (PDF) [file pone.0194667.s001.pdf]

Figure S2

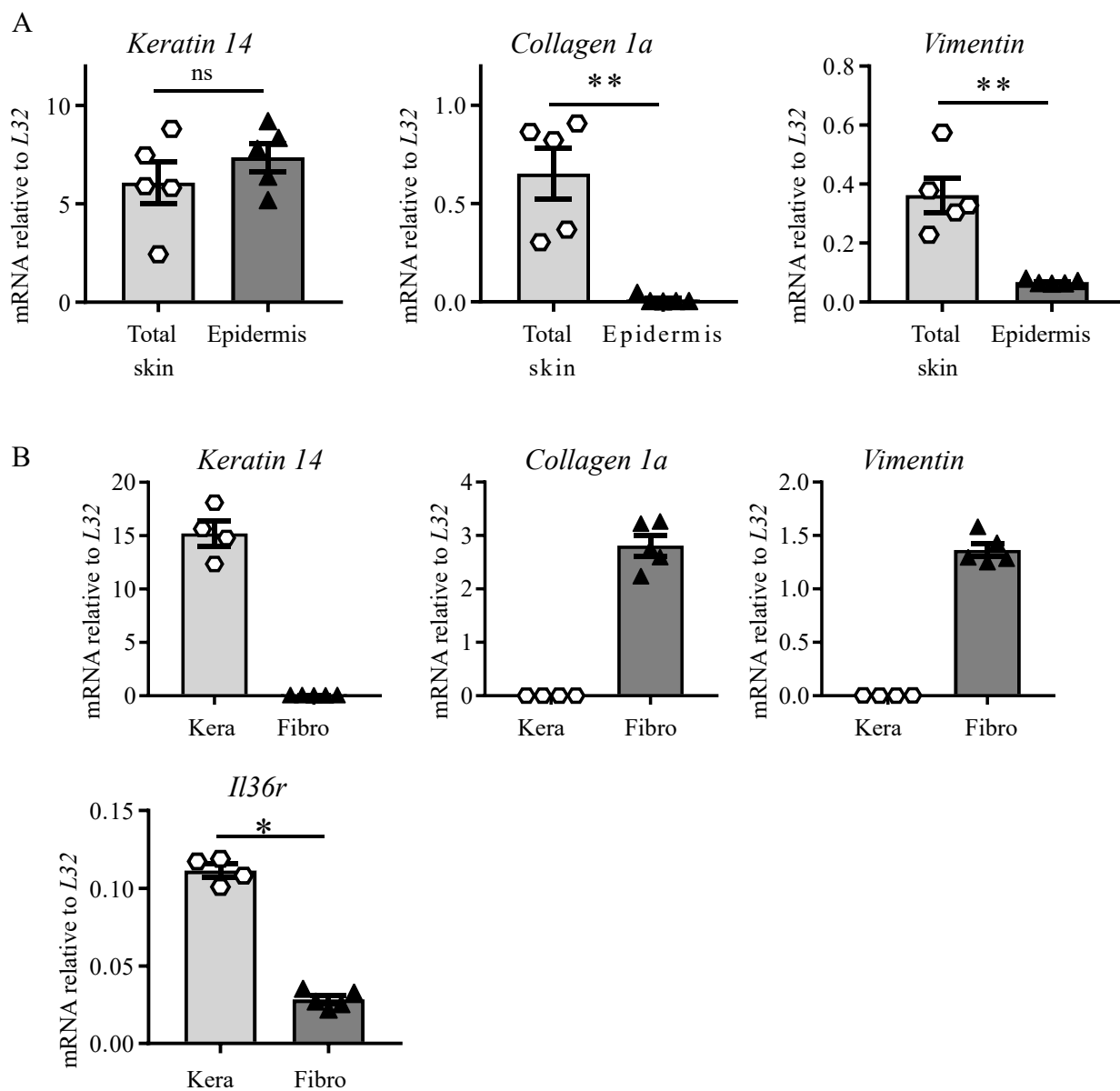

Supplement: S2 Fig — Basal mRNA expression of keratinocyte-specific Keratin 14, as well as of fibroblast-specific Collagen 1a and Vimentin was quantified by real-time RT-qPCR in total skin (n = 5) and epidermis (n = 5) of naïve BALB/c WT mice (A). Keratin 14, Collagen 1a, Vimentin, and Il-36r mRNA levels were quantified by real-time RT-qPCR in cultured primary skin keratinocytes (Kera, n = 4 independent cultures) and dermal fibroblasts (Fibro, n = 5 independent cultures) isolated from the skin of naïve WT BALB/c mice (B). Data are expressed relative to L32 levels. Results represent individual values and mean ± SEM. Statistical analysis was performed using an unpaired Mann-Whitney comparison test. A p-value < 0.05 was considered significant. *** p<0.001, ** p<0.01, * p<0.05. (PDF) [file pone.0194667.s002.pdf]

Figure S3

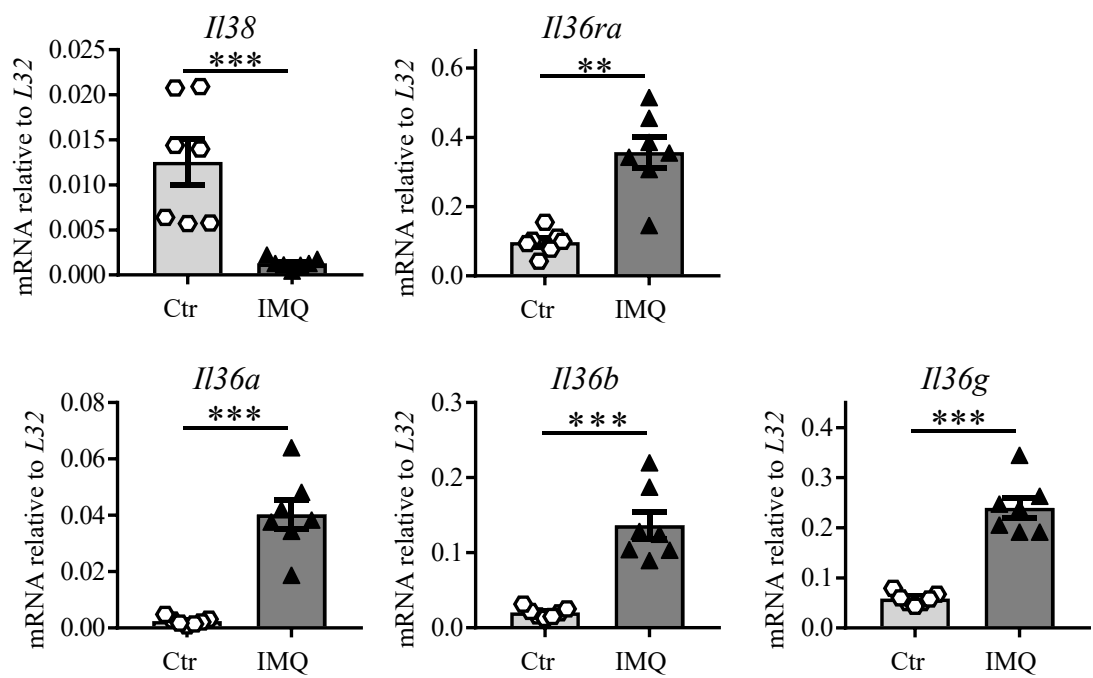

Supplement: S3 Fig — WT BALB/c mice were treated daily with a topical dose of 12.5mg of Aldara™ cream (0.625mg IMQ) for 8 days (n = 7). Skin mRNA levels for Il-38, Il-36ra, Il-36α, Il-36β and Il-36γ were quantified by real-time RT-qPCR in the non-treated ear (Ctr) and in the IMQ-treated ear on day 8. Data were expressed relative to L32 levels. Results represent individual values and mean ± SEM. Statistical analysis was performed by unpaired Mann-Whitney comparison test. A p-value < 0.05 was considered significant. ** p<0.01, *** p<0.001. (PDF) [file pone.0194667.s003.pdf]

Figure S4

A HE

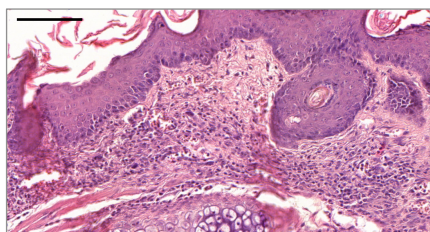

anti-Ly6G

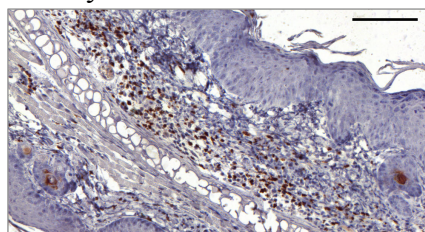

anti-CD3

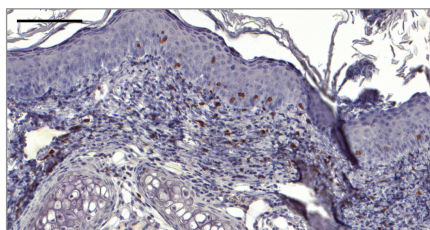

anti-B220

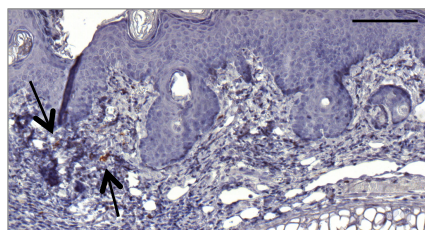

B anti-Ly6G

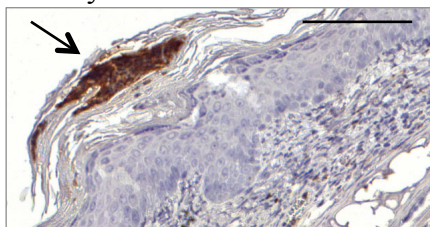

HE

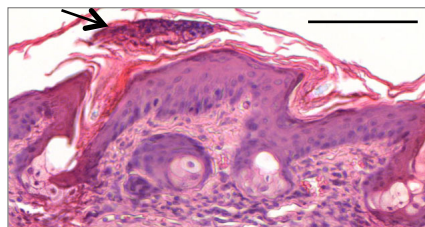

C anti-Ly6G

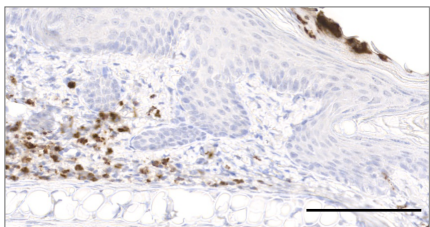

anti-CD3

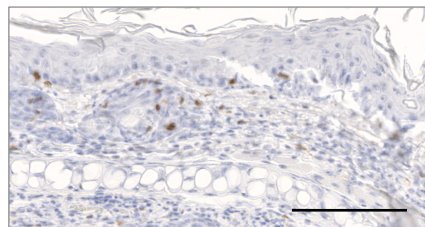

WT

anti-Ly6G

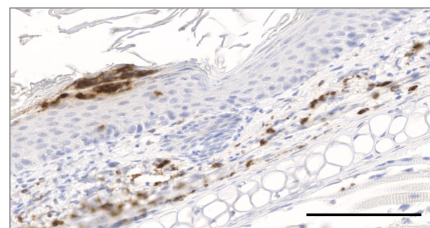

anti-CD3

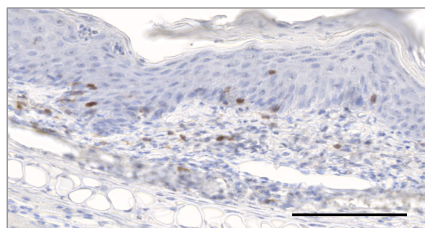

*Il-38*<sup>-/-</sup>

Supplement: S4 Fig — Representative HE (upper left panel), anti-Ly6G (brown staining, upper right panel), anti-CD3 (brown staining, lower left panel), and anti-B220 (brown staining, arrows, lower right panel) stained sections are shown for IMQ-treated WT ears at the peak of inflammation on day 7 (A). Representative anti-Ly6G (brown staining, left panel) and HE (right panel) stained sections including neutrophil-filled abscess-like structures located just beneath the stratum corneum (arrows) are shown for IMQ-treated WT ears at the peak of inflammation on day 7 (B). Representative anti-Ly6G (brown staining, left panels) and anti-CD3 (brown staining, right panels) stained sections are shown for IMQ-treated ears of WT (upper panels) or Il-38-/- (lower panels) littermate mice at the peak of inflammation on day 7 (C). Scale bar = 100 μM. (PDF) [file pone.0194667.s004.pdf]

Figure S5

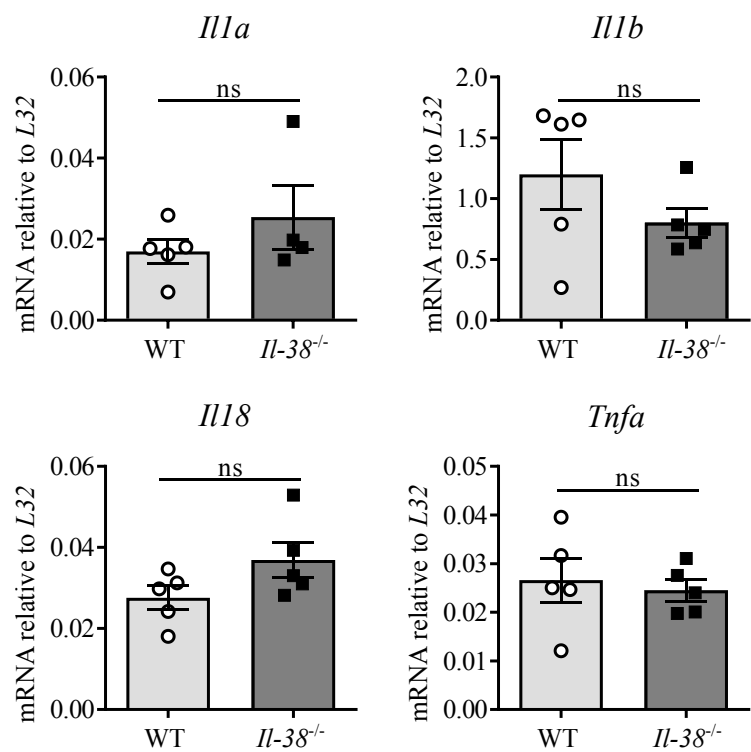

Supplement: S5 Fig — Il-38-/- mice and WT littermates were treated daily with a topical dose of 12.5mg of Aldara™ cream (0.625mg IMQ), for 7 days (n = 5). Skin mRNA levels for Il-1α, Il-1β, Il-18 and Tnfa were quantified by real-time RT-qPCR on day 7. Data were expressed relative to L32 levels. Results represent individual values and mean ± SEM. Statistical analysis was performed by unpaired Mann-Whitney comparison test. No significant differences were observed between the groups. (PDF) [file pone.0194667.s005.pdf]
